# Effect of Long-Term Sodium Hypochlorite Cleaning on Silicon Carbide Ultrafiltration Membranes Prepared via Low-Pressure Chemical Vapor Deposition

**DOI:** 10.3390/membranes14010022

**Published:** 2024-01-15

**Authors:** Asif Jan, Mingliang Chen, Michiel Nijboer, Mieke W. J. Luiten-Olieman, Luuk C. Rietveld, Sebastiaan G. J. Heijman

**Affiliations:** 1 Section of Sanitary Engineering, Department of Water Management, Faculty of Civil Engineering and Geosciences, Delft University of Technology, Stevinweg 1, 2628 CN Delft, The Netherlandsl.c.rietveld@tudelft.nl (L.C.R.); s.g.j.heijman@tudelft.nl (S.G.J.H.); 2 Inorganic Membranes, MESA + Institute for Nanotechnology, University of Twente, 7500 AE Enschede, The Netherlandsm.w.j.luiten@utwente.nl (M.W.J.L.-O.)

**Keywords:** silicon carbide, ultrafiltration, low-pressure chemical vapor deposition, sodium hypochlorite, chemical aging

## Abstract

Sodium hypochlorite (NaClO) is widely used for the chemical cleaning of fouled ultrafiltration (UF) membranes. Various studies performed on polymeric membranes demonstrate that long-term (>100 h) exposure to NaClO deteriorates the physicochemical properties of the membranes, leading to reduced performance and service life. However, the effect of NaClO cleaning on ceramic membranes, particularly the number of cleaning cycles they can undergo to alleviate irreversible fouling, remains poorly understood. Silicon carbide (SiC) membranes have garnered widespread attention for water and wastewater treatment, but their chemical stability in NaClO has not been studied. Low-pressure chemical vapor deposition (LP-CVD) provides a simple and economical route to prepare/modify ceramic membranes. As such, LP-CVD facilitates the preparation of SiC membranes: (a) in a single step; and (b) at much lower temperatures (700–900 °C) in comparison with sol-gel methods (ca. 2000 °C). In this work, SiC ultrafiltration (UF) membranes were prepared via LP-CVD at two different deposition temperatures and pressures. Subsequently, their chemical stability in NaClO was investigated over 200 h of aging. Afterward, the properties and performance of as-prepared SiC UF membranes were evaluated before and after aging to determine the optimal deposition conditions. Our results indicate that the SiC UF membrane prepared via LP-CVD at 860 °C and 100 mTorr exhibited excellent resistance to NaClO aging, while the membrane prepared at 750 °C and 600 mTorr significantly deteriorated. These findings not only highlight a novel preparation route for SiC membranes in a single step via LP-CVD, but also provide new insights about the careful selection of LP-CVD conditions for SiC membranes to ensure their long-term performance and robustness under harsh chemical cleaning conditions.

## 1. Introduction

Silicon carbide (SiC) ultrafiltration (UF) membranes have found widespread applications in industrial wastewater [1,2,3], produced water [4], grey water [5], and surface water treatment [6]. In addition, they are employed as a pre-treatment step prior to reverse-osmosis [7] and for pool water filtration in public swimming pools [8]. The popularity of SiC UF membranes stems from their ability to provide: (i) high permeate fluxes due to their hydrophilic nature; (ii) a high-quality permeate irrespective of variation in feed quality; (iii) an excellent hydrothermal stability; (iv) a good stability against pressure and pH gradients; and (v) a high porosity and uniform pore size distribution [9,10,11]. Furthermore, various preparation methods are available to obtain SiC membranes, namely, sol-gel, and vapor deposition methods [12]. In general, sol-gel methods require multiple coating cycles of the desired material and high-temperature sintering, which makes the process energy-intensive [13,14]. In contrast, LP-CVD provides a straightforward method to prepare SiC membranes without the sintering step. 

Wastewaters consist of a complex mixture of contaminants, including organic matter and colloidal particles [15,16]. These contaminants are susceptible to attaching to the membrane surface via physisorption or chemisorption. In both cases, a significant decline in flux and an increase in trans-membrane pressure (TMP) have been observed [17,18,19]. If the physisorption of the contaminants can be alleviated by back-washing the membrane, the fouling is called reversible. However, if the contaminants are chemisorbed on the membrane surface, the fouling is irreversible and chemical cleaning must be performed to remove the contaminants and restore membrane flux. Chemical cleaning is performed using an aggressive oxidizing chemical, e.g., sodium hypochlorite (NaClO), and repetitive chemical cleaning results in the degradation of membranes of both organic and inorganic nature [20,21,22]. Consequentially, the permeability and selectivity of the membrane are compromised.

From the perspective of organic membranes, various studies were performed on different polymeric membranes (polyethersulphone, polyvinylpyrrolidone, polyvinylchloride) to study the degradation of membrane properties upon NaClO exposure and the underlying mechanisms responsible for degradation. Susanto et al. observed an increase in pure water flux of polyethersulphone (PES) membranes after exposure to NaClO [23]. Mechanistic analysis of the degradation of PES membranes has also been reported in various studies, and it was concluded that the chain scission of PES is responsible for altering membrane properties [24,25]. In comparison, from the perspective of inorganic membranes, studies on the effect of membrane properties by NaClO exposure are scarce. Kramer et al. tested the chemical stability of ceramic ultrafiltration and nanofiltration membranes by exposing them to 1% NaClO solution for 100 h. It was observed that the NaClO deteriorated the glass seal at the edges of the membrane, and an increase in water permeability and molecular weight cut-off was observed. However, mechanistic insights were not provided [21]. 

NaClO is commonly employed for the chemical cleaning of membranes. The highly oxidative nature of NaClO can selectively leach out organic, inorganic, and biological fouling, and restore the primary properties of the membrane [26]. The active species, present in a NaClO solution depend on the pH of the solution. NaClO dissociates into hypochlorous acid (HOCl) and hypochlorite ion (^−^OCl) depending on the pH. HOCl is the active species in germicidal activity, and the concentration of ^−^OCl determines the cleaning efficiency [27]. The concentration of ^−^OCl is the highest in the pH range of 10–12 [28]. Therefore, chemical cleaning is usually performed within the aforementioned pH range. However, these active species can also deteriorate the physicochemical properties of the selective layer of the membrane, and the service life of a membrane is then subject to the total hours of chemical cleaning with NaClO. In this regard, various studies on polymeric membranes have been performed to show that the long-term effects of NaClO cleaning on the membrane properties are detrimental [29,30,31]. However, to the authors’ knowledge, studies on the impact of long-term NaClO aging on SiC UF membranes are still lacking.

Therefore, in the present work, SiC UF membranes were prepared via LP-CVD under two different deposition conditions. Subsequently, the effects of accelerated NaClO aging on the physiochemical properties and performance of SiC UF membranes were studied to determine the suitable LP-CVD deposition conditions. The chemical robustness of the SiC UF membranes was investigated by exposing tubular SiC UF membranes to 5% NaClO for 200 h to simulate long-term aging. The surface chemical composition, surface morphology, pore size, and water permeability of the pristine and aged SiC UF membranes were scrutinized to gauge the chemical robustness of the as-prepared SiC UF membranes. 

## 2. Materials and Methods

### 2.1. Materials and Chemical Agents

**Figure 1:**
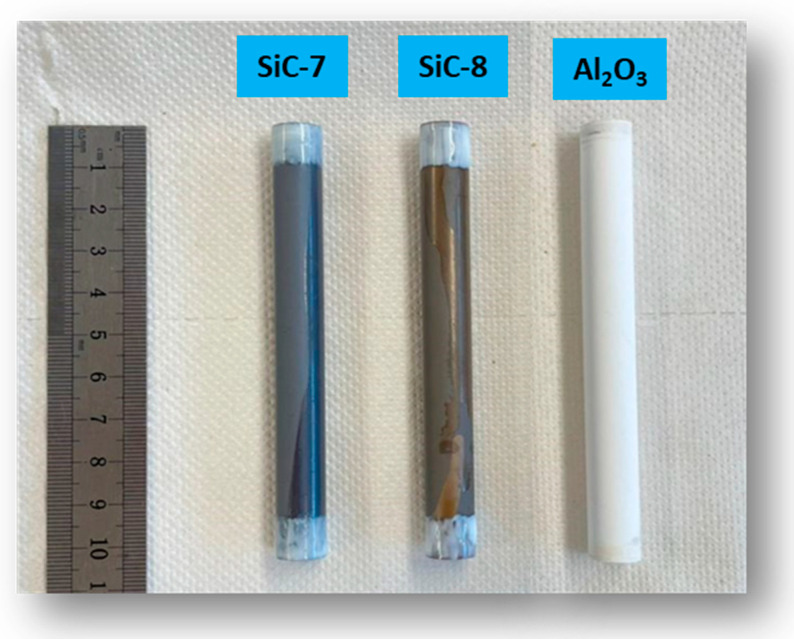
Sealed pristine alumina and SiC-coated membranes.

Commercial tubular alumina membranes, obtained from CoorsTek, the Netherlands, were used as substrates for LP-CVD of SiC. The membranes had an inner diameter of 7 mm, an outer diameter of 10 mm, and were 10 cm long (Figure 1). As per suppliers’ specifications, the membranes consisted of a 100 nm alumina selective layer on a 600 nm macroporous alumina support. Great variations were observed for the water permeability of the membranes [32]. Therefore, membranes with a water permeability of ca. 350 L.m^−2^.h^−1^.bar^−1^ were selected for the LP-CVD of SiC.

Commercially available 12.5% sodium hypochlorite (NaClO) was purchased from Sigma-Aldrich Chemicals (The Netherlands). Then, 5% NaClO was prepared by diluting the 12.5% stock solution, and the pH was maintained at 12. Deionized water was used to prepare all the solutions.

### 2.2. Low-Pressure Chemical Vapor Deposition

A hot-wall LP-CVD furnace (Tempress Systems BV, The Netherlands) was used for the deposition of SiC. The construction of the LP-CVD system is outlined elsewhere [32]. The precursors used were dichlorosilane (SiH_2_Cl_2_) and 5% acetylene (C_2_H_2_) in hydrogen (H_2_) balance for the silicon (Si) and carbon © sources, respectively. Ultrapure nitrogen (N_2_) from a liquid N_2_ source was employed as a purging gas in the system. During SiC deposition, the membranes were placed longitudinally to the flow of precursor gases.

Deposition conditions were adapted from the study of Morana et al. to obtain a thin amorphous SiC layer on the membrane surface [33], and the SiC deposition was performed at two different temperatures and pressures. Due to difficulties in directly measuring the growth rate of SiC in the pores of the membrane, the SiC growth rate at both conditions was measured on silicon wafers with ellipsometry. Low-temperature SiC deposition (SiC-7) was carried out at a temperature of 750 °C, pressure of 600 mTorr, and deposition time of 60 min. High-temperature SiC deposition (SiC-8) was carried out at a temperature of 860 °C, pressure of 100 mTorr, and deposition time of 30 min. Both deposition conditions led to the same thickness of the SiC layer on silicon wafers (Supplementary Table S1). The temperature, pressure, and deposition time were selected so that a SiC-selective layer of the same thickness was obtained for membranes. 

### 2.3. Membrane Accelerated Aging Procedure

Before the aging experiments, the SiC-7 and SiC-8 membranes were soaked in ultrapure water for 24 h, and afterward, their water permeability was measured. For the membrane aging procedure, dried membrane samples were soaked in a 5% NaClO solution in an air-tight container at an ambient temperature (25 ± 3 °C) in the dark for 200 h. This corresponds to an exposure dose of 10,000 g.h/L. The aging solutions were replaced every 24 h to avoid variation in concentration and pH with time. The aged SiC-7 and SiC-8 membranes are referred to as SiC-7-2A and SiC-8-2A, respectively. After 200 h, SiC-7-2A and SiC-8-2A were removed from the 5% NaClO solution, rinsed with ultrapure water, and soaked in ultrapure water overnight to remove residual NaClO species before characterization and performance analysis.

### 2.4. Membrane Characterization and Performance Evaluation 

The morphology of the pristine alumina, the SiC-7/SiC-8, and the SiC-7-2A/SiC-8-2A membranes was observed via scanning electron microscopy (SEM, FEI Nova NanoSEM 450, USA). An energy-dispersive X-ray (EDX) analyzer coupled with SEM was used to determine the Si atomic percentage. Sample preparation for SEM involved breaking the membranes with a hammer to obtain a flat specimen, which was afterward sputter-coated with gold to increase sample conductivity to achieve clear images. 

The surface chemical composition of the SiC-7/SiC-8 and SiC-7-2A/SiC-8-2A membranes was evaluated via X-ray photoelectron spectroscopy (XPS). XPS spectra were obtained using a ThermoFisher K-alpha XPS system. Further processing of the XPS spectra was achieved using CasaXPS software (Version 2.3.25).

The average pore size of the membranes was measured via capillary flow porometry (Porolux 500, IBFT GmbH, Germany). FC43 (Benelux Scientific B.V., The Netherlands) was used as a wetting agent for porometry measurements, and flow and feed pressure were recorded in time. The pore size was then calculated using the Young–Laplace equation [34]:

D=(4γ.cos⁡θ.SF)/P

where *D* is the pore diameter of the membrane (m), *γ* is the surface tension of the wetting liquid (N/m), *θ* is the contact angle of the liquid on the membrane surface (0°), *P* is the used pressure (bar), and *SF* is the shape factor (*SF* is 1 based on the assumption that all pores have an exact cylindrical shape). 

**Figure 2:**
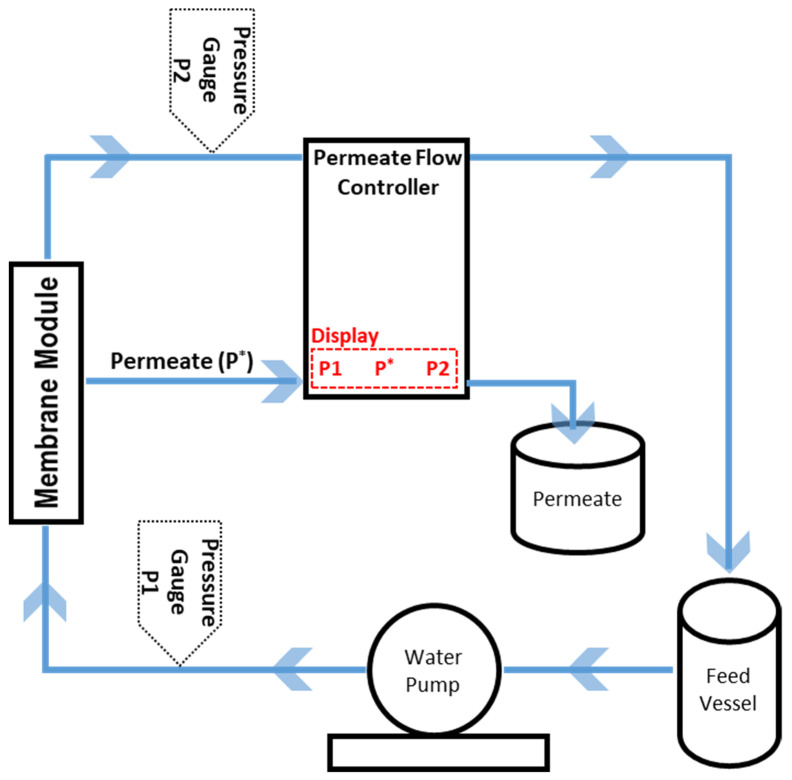
Water permeability measurement setup. P* is permeate flow rate.

The pure water permeability of the membranes was measured under a constant flux in an in-house built cross-flow filtration setup for tubular membranes using deionized water (Figure 2). 

## 3. Results and Discussion

### 3.1. Microstructure and Surface Composition of the SiC Membranes

**Figure 3:**
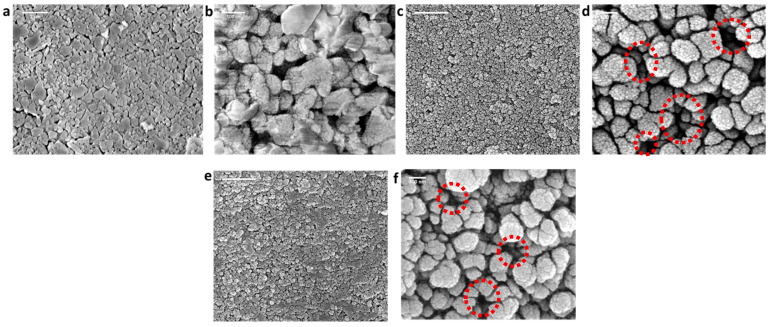
Surface morphology analysis via SEM of (**a**,**b**) the pristine alumina membrane; (**c**,**d**) SiC-7 membrane; and (**e**,**f**) SiC-8 membrane. Red circles highlight the difference in porosities.

Surface morphologies of the pristine (alumina) and SiC-deposited membranes (SiC-7, SiC-8) were analyzed via SEM, as shown in Figure 3. The macroporous alumina membrane comprised randomly oriented fine and coarse particles (Figure 3a,b). The discrepancy in shape and size of the alumina particles resulted in a non-homogeneous surface. The surface of SiC-7 membrane comprised amorphous SiC nodules, and a decrease in porosity was observed in comparison with the alumina membrane (Figure 3c,d). The SiC deposition at 860 °C resulted in a continuous dense SiC layer formation [35], and the decrease in porosity was larger than that observed for SiC-7 (Figure 3e,f). Our observations are consistent with the LP-CVD growth mechanisms reported previously, i.e., when SiC is deposited on a foreign substrate (which, in this case, is Al_2_O_3_), the growth proceeds via the three-dimensional Volmer–Weber island growth mechanism [36]. At the low-deposition temperature of 750 °C, the growth kinetics were slow, leading to the formation of distinct SiC islands, which subsequently grow into SiC nodules, as suggested by Greene [36]. However, at a high deposition temperature of 860 °C, fast growth kinetics led to the coalescing and growth of SiC nodules to form a homogeneous and continuous layer [37,38]. The intrinsic stress, introduced due to either deposition conditions or mismatch in thermal coefficients between the substrate and deposited material, is greatly reduced by the transition in morphology of SiC from nodules to a continuous layer [39]. Consequently, the growth of a continuous SiC layer will properly shield the Al_2_O_3_ particles, and hence the interfacial energy between Al_2_O_3_ substrate and a SiC layer will be minimized [40], leading to the enhancement of adhesion strength between the SiC layer and Al_2_O_3_ substrate. 

**Figure 4:**
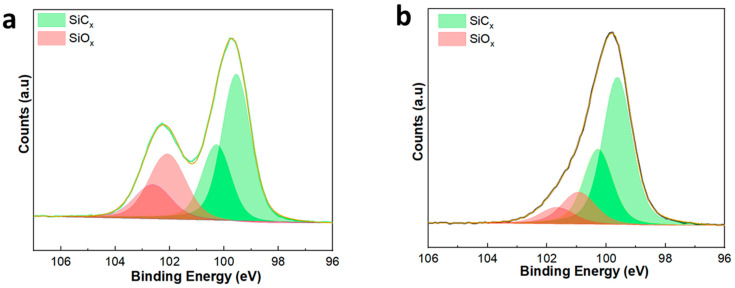
XP-spectra of (**a**) the SiC-7 membrane; and (**b**) the SiC-8 membrane.

XPS was performed to study the elemental makeup of the SiC membrane surface. Figure 4a,b presents the Si *2p* XPS spectra of the SiC-7 and SiC-8 membranes. In both cases, the Si *2p* peaks were deconvoluted into four peaks, confirming the presence of Si, both in the form of oxides (SiO_x_) and carbides (SiC_x_) [41,42]. The presence of the Si-C4 peak at a binding energy of ~99.5 in Figure 4a, and the presence of the Si-C4 peak at a binding energy of ~99.6 in Figure 4b confirm the deposition of SiC via LP-CVD [43]. The presence of Si-C3-O, Si-C2-O2/Si-C-O3, and Si-O4 suggests that the SiC is of amorphous nature.

**Figure 5:**
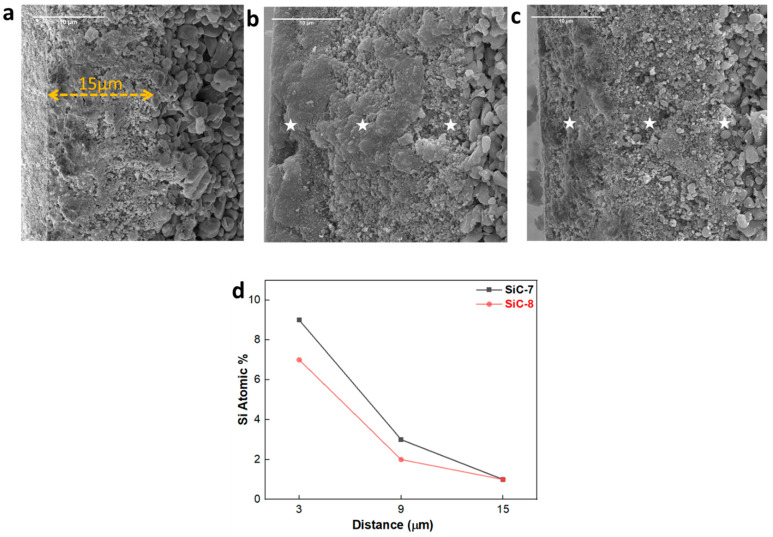
SEM cross-sectional images of (**a**) the pristine membrane; (**b**) SiC-7 membrane; (**c**) SiC-8 membrane; and (**d**) EDX spectra.

The morphological transformation of the cross-section of the membranes after LP-CVD of SiC was further studied by SEM-EDX. The pristine alumina membrane had an alumina separation layer ca. 15 µm in thickness, and it had a granular appearance (Figure 5a). After the deposition of SiC at 750 °C, huge deposits of nodular SiC can be observed on the surface of the membrane (Figure 5b). Additionally, random deposits of SiC were also observed along the cross-section of the membrane. Since ceramic material has two types of pores, i.e., (i) open pores, which are accessible to precursors; and (ii) closed pores, which are not accessible to precursors [44], the occurrence of haphazard deposits of SiC along the cross-section of the membrane, deposited at 750 °C, can be explained. However, as shown in Figure 5c, the deposition of SiC at 860 °C resulted in the formation of a thin SiC layer at the surface of the membrane, and deposits of SiC were also observed at the sub-surface (3–6 µm) of the membrane. Additionally, Si deposits were observed by EDX only until the cross-sectional depth of 9 µm for both SiC-7 and SiC-8 (Figure 5d). Hence, it was concluded that the newly formed SiC selective layer did not completely shield the previous alumina-selective layer ca. 15 µm in thickness. In fact, the newly formed SiC-selective layer, in both cases, had a thickness of ca. 9 µm. Furthermore, the difference in cross-sectional morphology between SiC-7 and SiC-8 membranes can be explained by the difference in deposition conditions. At 750 °C, the high pressure and high concentration of the precursor gases probably led to a decrease in the mean free path of the precursor gases [45]. Therefore, the deposition proceeded via both gas phase reactions of the precursors and reactions of the adsorbed precursors at the surface of the membrane [46], which is confirmed by the high atomic percentage of Si on the surface of the membrane shown in Figure 5d. At 860 °C, the pressure and concentration of precursor gases was relatively low, which only led to the deposition of a dense SiC via reactions of adsorbed precursors on the surface of the membrane. Different pressures were chosen at the two respective deposition temperatures in order to elucidate the effect of pressure on the penetration depth of precursors. However, it can be seen in Figure 5d that the chamber pressure did not affect the penetration depth of precursors.

### 3.2. Effect of SiC LP-CVD and NaClO Aging on Membrane Permeability and Pore Size 

**Figure 6:**
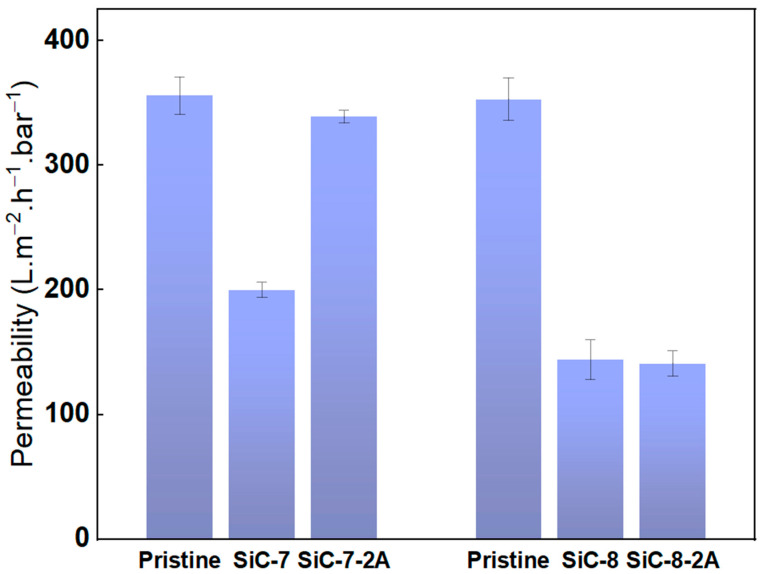
Pure water permeability of the pristine; SiC-7; SiC-8; SiC-7-2A; and SiC-8-2A membranes.

Pure water permeability of the pristine SiC-7/SiC-7-2A, and SiC-8/SiC-8-2A was measured and the results are shown in Figure 6. The pure water permeability of the pristine alumina membranes was ca. 350 L.m^−2^.h^−1^.bar^−1^, while for the SiC-7 membrane, it dropped to 200 L.m^−2^.h^−1^.bar^−1^. Increasing the SiC deposition temperature to 860 °C led to a significant reduction in pure water permeability of the SiC-8 membrane to 128 L.m^−2^.h^−1^.bar^−1^. The difference between SiC-7 and SiC-8 membranes can be explained by the difference in morphology of the SiC, as described earlier. In their study, Alam et al. concluded that in contrast to a smooth surface, a rough surface has a higher specific surface area. As a consequence, more surface sites will be available for the water molecules and thus water permeability will be higher [47], which is consistent with our findings. Aging in 5% NaClO for 200 h had different effects on the pure water permeability of both membranes. The pure water permeability of SiC-7-2A membrane increased to ca. 339 L.m^−2^.h^−1^.bar^−1^, and the membrane changed its appearance from black to off-white after aging, indicating that the SiC was deteriorated (Figure S1). In contrast, the pure water permeability of the SiC-8-2A membrane was unaffected by NaClO aging.

**Figure 7:**
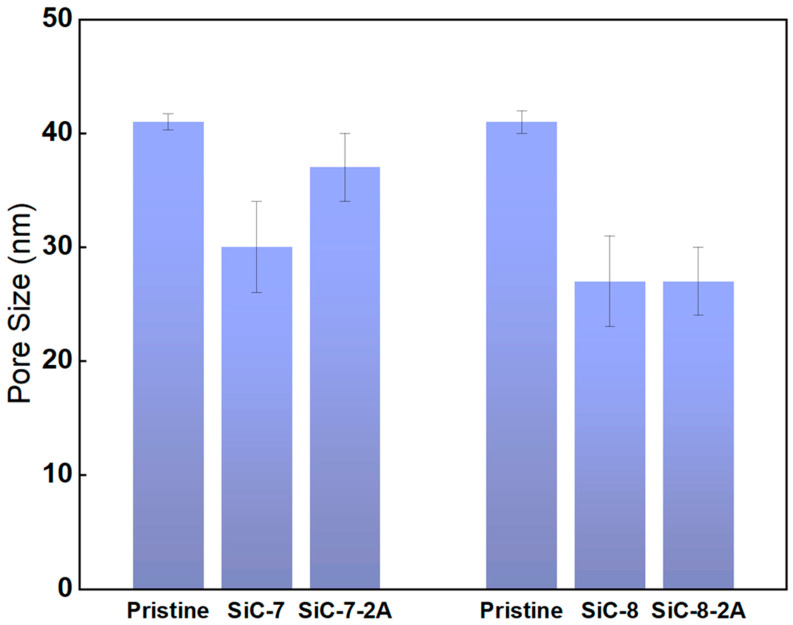
Pore size measurements of the pristine; SiC-7; SiC-8; SiC-7-2A; and SiC-8-2A membranes.

Pore size measurements were conducted to obtain insights in the pore size distribution of the membranes after SiC deposition and NaClO aging (Figure 7). The actual mean pore size of the pristine membrane was determined to be 42 nm, which differed greatly from the supplier’s claimed pore size of 100 nm. The pristine membrane had a broad pore size distribution with the smallest pores of ca. 26 nm and the largest pores of ca. 98 nm. The mean pore size dropped to ca. 30 nm for the SiC-7 membrane, and it was reduced to ca. 27 nm for the SiC-8 membrane. The difference in pore size confirms the difference in pure water permeability. After aging, the mean pore size of the SiC-7-2A membrane increased to ca. 37 nm, which is in accordance with the SEM images, showing the deterioration of the SiC-selective layer. In contrast, the mean pore size of SiC-8-2A membrane was unaffected by aging, confirming the chemical robustness of the SiC-8 membrane in NaClO.

### 3.3. Morphological and Chemical Post-Mortem Analysis of NaClO-Aged SiC Membranes

**Figure 8:**
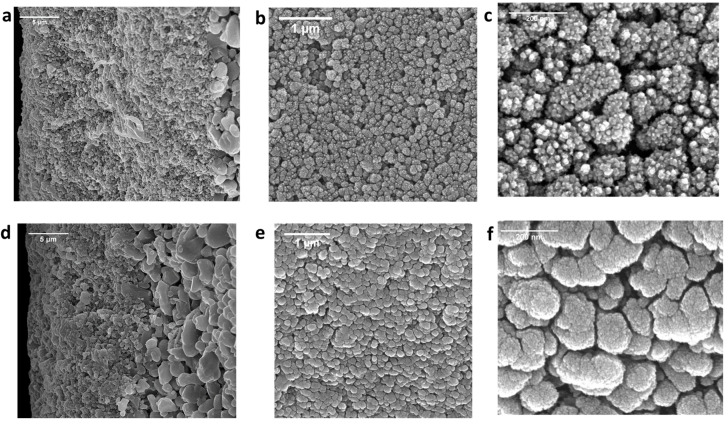
SEM cross-sectional images of (**a**) the SiC-7-2A membrane; and (**d**) SiC-8-2A membrane. SEM surface images of (**b**,**c**) the SiC-7-2A membrane; and (**e**,**f**) SiC-8-2A membrane.

The effects of NaClO aging on the surface and cross-sectional morphology of the SiC membranes were studied via SEM. The SEM images of SiC-7-2A and SiC-8-2A membranes are presented in Figure 8a–f. It can be observed from Figure 8a–c that the SiC nodules on the surface and random deposits of SiC along the cross-section of SiC-7-2A were removed by the exposure to NaClO. The removal of the SiC-selective layer led to the increase in porosity and pore size of SiC-7-2A (Figure 7), consequentially water permeability of SiC-7-2A increased (Figure 6). For SiC-8-2A, NaClO exposure had no effect on the SiC-selective layer (Figure 8d–f). As such, water permeability (Figure 6) and pore size (Figure 7) remained the same. These results confirm the stability of SiC layer deposited at a high temperature (860 °C) toward harsh oxidizing treatment of NaClO. We speculate that the deposition of SiC on the alumina substrate at a high temperature (860 °C) and low pressure (100 mTorr) led to the formation of a continuous and dense SiC layer [48]. Therefore, the ^−^OCl species could not corrode the SiC layer. On the other hand, SiC deposition at a low temperature (750 °C) and high pressure (600 mTorr) led to the formation of porous and amorphous SiC nodules. Consequentially, the ^−^OCl species completely corroded the SiC-selective layer. However, further research is warranted to provide a comprehensive understanding of the mechanisms underlying deterioration.

**Figure 9:**
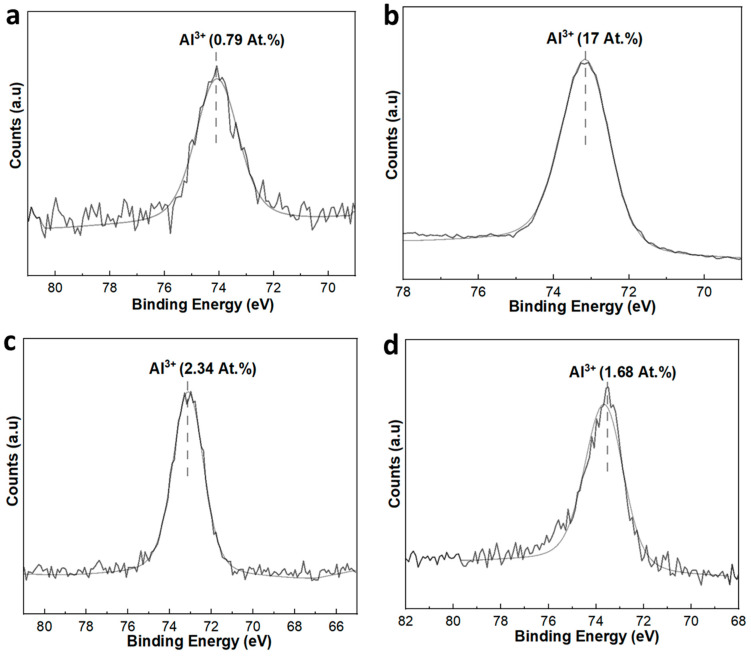
XP-spectra of SiC-7 and SiC-7-2A membranes (**a**,**b**); and SiC-8 and SiC-8-2A membranes (**c**,**d**).

Al *2p* spectra of unaged and aged membranes were also analyzed, and the percentage atomic concentration (at.%) of Al^3+^ was calculated by the area under the peaks to gain insights into the surface composition of the membranes (Figure 9). For the SiC-7 membrane, the at.% of Al^3+^ was 0.79 (Figure 9a). However, after NaClO aging, the at.% of Al^3+^ in SiC-7-2A membrane increased to 17 (Figure 9b). This confirms that the SiC layer was removed, and the surface now consisted of Al_2_O_3_ particles. These results are consistent with the SEM results (Figure 8). For the SiC-8 and SiC-8-2A membranes, the at.% of Al^3+^ was 2.34 and 1.68, respectively, which shows that the Al_2_O_3_ particles were still shielded by a SiC layer, even after aging in NaClO (Figure 9c,d).

## 4. Conclusions

SiC UF membranes were prepared under two different deposition conditions via LP-CVD. As-prepared SiC membranes were aged in NaClO for 200 h. Afterward, the effect of aging on the properties and performance of the SiC membranes was studied.

SEM images coupled with EDX spectra of as-prepared membranes showed that a ca. 9 µm thick SiC-selective layer was formed after LP-CVD. Under high-temperature deposition conditions (860 °C and 100 mTorr), a continuous film of a SiC-selective layer was obtained. On the other hand, under low-temperature deposition conditions (750 °C and 600 mTorr), SiC was deposited in the form of amorphous nodules. Pure water permeability and pore size measurements revealed that the SiC deposition at a high temperature led to the formation of a robust SiC-selective layer which was stable in NaClO for 200 h. Additionally, XPS and SEM results demonstrated that SiC deposited at a low temperature completely deteriorated after 200 h of NaClO exposure. 

Although further study will be required to elucidate the underlying mechanism explaining why the high-temperature LP-CVD conditions lead to continuous, highly adhesive, and stable SiC film, it is expected that the findings in this study possess significant implications for the preparation of chemically robust SiC UF membranes in a single step, without the need for sintering, and could be an alternative to full SiC UF membranes. 

## Data Availability

Data are contained within the article.

## Supplementary Materials

The following supporting information can be downloaded at: https://www.mdpi.com/article/10.3390/membranes14010022/s1, Figure S1: SiC membranes after ageing in NaClO for 200hrs. Table S1: SiC layer thickness as a function of different deposition conditions.

## References

[1] Fraga M.C., Sanches S., Crespo J.G., Pereira V.J. (2017). Assessment of a New Silicon Carbide Tubular Honeycomb Membrane for Treatment of Olive Mill Wastewaters. Membranes.

[2] Karimzadeh S., Safaei B., Jen T.-C., Oviroh P.O. (2021). Enhanced removal efficiency of heavy metal ions from wastewater through functionalized silicon carbide membrane: A theoretical study. J. Water Process Eng..

[3] Lanjewar T., Badwaik P., Varma M.N. (2021). Removal of water from the spent mixture of nitric- sulfuric acid by using silicon carbide ceramic diffusive membrane. Sep. Purif. Technol..

[4] Fraga M.C., Sanches S., Pereira V.J., Crespo J.G., Yuan L., Marcher J., de Yuso M.V.M., Rodríguez-Castellón E., Benavente J. (2017). Morphological, chemical surface and filtration characterization of a new silicon carbide membrane. J. Eur. Ceram. Soc..

[5] Das D., Baitalik S., Haldar B., Saha R., Kayal N. (2017). Preparation and characterization of macroporous SiC ceramic membrane for treatment of waste water. J. Porous Mater..

[6] Das D., Kayal N., Marsola G.A., Damasceno L.A., Innocentini M.D.d.M. (2020). Permeability behavior of silicon carbide-based membrane and performance study for oily wastewater treatment. Int. J. Appl. Ceram. Technol..

[7] Nejati S., Mirbagheri S.A., Warsinger D.M., Fazeli M. (2019). Biofouling in seawater reverse osmosis (SWRO): Impact of module geometry and mitigation with ultrafiltration. J. Water Process Eng..

[8] Neufert R., Moeller M., Bakshi A.K., Narayan R., Colombo P., Kirihara S., Widjaja S. (2013). Dead-End Silicon Carbide Micro-Filters for Liquid Filtration. Advances in Bioceramics and Porous Ceramics VI.

[9] Elyassi B., Sahimi M., Tsotsis T.T. (2007). Silicon carbide membranes for gas separation applications. J. Membr. Sci..

[10] Eray E., Candelario V.M., Boffa V., Safafar H., Østedgaard-Munck D.N., Zahrtmann N., Kadrispahic H., Jørgensen M.K. (2021). A roadmap for the development and applications of silicon carbide membranes for liquid filtration: Recent advancements, challenges, and perspectives. Chem. Eng. J..

[11] Hotza D., Di Luccio M., Wilhelm M., Iwamoto Y., Bernard S., Diniz da Costa J.C. (2020). Silicon carbide filters and porous membranes: A review of processing, properties, performance and application. J. Membr. Sci..

[12] Wang Q., Zhou R., Tsuru T. (2022). Recent Progress in Silicon Carbide-Based Membranes for Gas Separation. Membranes.

[13] Shi S., Jian K., Fang M., Guo J., Rao P., Li G. (2023). SiO2 Modification of Silicon Carbide Membrane via an Interfacial In Situ Sol–Gel Process for Improved Filtration Performance. Membranes.

[14] Wang Q., Yu L., Nagasawa H., Kanezashi M., Tsuru T. (2020). Tuning the microstructure of polycarbosilane-derived SiC(O) separation membranes via thermal-oxidative cross-linking. Sep. Purif. Technol..

[15] Huang M.-h., Li Y.-m., Gu G.-w. (2010). Chemical composition of organic matters in domestic wastewater. Desalination.

[16] Jungclaus G., Avila V., Hites R. (1978). Organic compounds in an industrial wastewater: A case study of their environmental impact. Environ. Sci. Technol..

[17] Chen M., Heijman S.G.J., Luiten-Olieman M.W.J., Rietveld L.C. (2022). Oil-in-water emulsion separation: Fouling of alumina membranes with and without a silicon carbide deposition in constant flux filtration mode. Water Res..

[18] Hofs B., Ogier J., Vries D., Beerendonk E.F., Cornelissen E.R. (2011). Comparison of ceramic and polymeric membrane permeability and fouling using surface water. Sep. Purif. Technol..

[19] Jin L., Ong S.L., Ng H.Y. (2010). Comparison of fouling characteristics in different pore-sized submerged ceramic membrane bioreactors. Water Res..

[20] Eray E., Candelario V.M., Boffa V. (2021). Ceramic Processing of Silicon Carbide Membranes with the Aid of Aluminum Nitrate Nonahydrate: Preparation, Characterization, and Performance. Membranes.

[21] Kramer F.C., Shang R., Scherrenberg S.M., Rietveld L.C., Heijman S.J.G. (2019). Quantifying defects in ceramic tight ultra- and nanofiltration membranes and investigating their robustness. Sep. Purif. Technol..

[22] Malczewska B., Zak A. (2019). Structural Changes and Operational Deterioration of the Uf Polyethersulfone (Pes) Membrane Due to Chemical Cleaning. Sci. Rep..

[23] Susanto H., Ulbricht M. (2009). Characteristics, performance and stability of polyethersulfone ultrafiltration membranes prepared by phase separation method using different macromolecular additives. J. Membr. Sci..

[24] Kourde-Hanafi Y., Loulergue P., Szymczyk A., Van der Bruggen B., Nachtnebel M., Rabiller-Baudry M., Audic J.-L., Pölt P., Baddari K. (2017). Influence of PVP content on degradation of PES/PVP membranes: Insights from characterization of membranes with controlled composition. J. Membr. Sci..

[25] Prulho R., Therias S., Rivaton A., Gardette J.-L. (2013). Ageing of polyethersulfone/polyvinylpyrrolidone blends in contact with bleach water. Polym. Degrad. Stab..

[26] Li K., Su Q., Li S., Wen G., Huang T. (2021). Aging of PVDF and PES ultrafiltration membranes by sodium hypochlorite: Effect of solution pH. J. Environ. Sci..

[27] Fukuzaki S. (2006). Mechanisms of Actions of Sodium Hypochlorite in Cleaning and Disinfection Processes. Biocontrol Sci..

[28] Luna-Trujillo M., Palma-Goyes R., Vazquez-Arenas J., Manzo-Robledo A. (2020). Formation of active chlorine species involving the higher oxide MO_x+1_ on active Ti/RuO_2_-IrO_2_ anodes: A DEMS analysis. J. Electroanal. Chem..

[29] Dibrov G., Kagramanov G., Sudin V., Grushevenko E., Yushkin A., Volkov A. (2020). Influence of sodium hypochlorite treatment on pore size distribution of polysulfone/polyvinylpyrrolidone membranes. Membranes.

[30] Ding J., Wang S., Xie P., Zou Y., Wan Y., Chen Y., Wiesner M.R. (2020). Chemical cleaning of algae-fouled ultrafiltration (UF) membrane by sodium hypochlorite (NaClO): Characterization of membrane and formation of halogenated by-products. J. Membr. Sci..

[31] Zhang Y., Wang J., Gao F., Tao H., Chen Y., Zhang H. (2017). Impact of sodium hypochlorite (NaClO) on polysulfone (PSF) ultrafiltration membranes: The evolution of membrane performance and fouling behavior. Sep. Purif. Technol..

[32] Chen M., Shang R., Sberna P.M., Luiten-Olieman M.W.J., Rietveld L.C., Heijman S.G.J. (2020). Highly permeable silicon carbide-alumina ultrafiltration membranes for oil-in-water filtration produced with low-pressure chemical vapor deposition. Sep. Purif. Technol..

[33] Morana B., Pandraud G., Creemer J.F., Sarro P.M. (2013). Characterization of LPCVD amorphous silicon carbide (a-SiC) as material for electron transparent windows. Mater. Chem. Phys..

[34] Shang R., Vuong F., Hu J., Li S., Kemperman A.J.B., Nijmeijer K., Cornelissen E.R., Heijman S.G.J., Rietveld L.C. (2015). Hydraulically irreversible fouling on ceramic MF/UF membranes: Comparison of fouling indices, foulant composition and irreversible pore narrowing. Sep. Purif. Technol..

[35] Racz A.S., Kerner Z., Nemeth A., Panjan P., Peter L., Sulyok A., Vertesy G., Zolnai Z., Menyhard M. (2017). Corrosion Resistance of Nanosized Silicon Carbide-Rich Composite Coatings Produced by Noble Gas Ion Mixing. ACS Appl. Mater. Interfaces.

[36] Greene J.E., Martin P.M. (2010). Chapter 12—Thin Film Nucleation, Growth, and Microstructural Evolution: An Atomic Scale View. Handbook of Deposition Technologies for Films and Coatings.

[37] Pashley D.W., Stowell M.J. (1966). Nucleation and Growth of Thin Films as Observed in the Electron Microscope. J. Vac. Sci. Technol..

[38] Pashley D.W., Stowell M.J., Jacobs M.H., Law T.J. (1964). The growth and structure of gold and silver deposits formed by evaporation inside an electron microscope. Philos. Mag..

[39] Carlsson J.-O., Martin P.M., Martin P.M. (2010). Chapter 7—Chemical Vapor Deposition. Handbook of Deposition Technologies for Films and Coatings.

[40] You Q., Xiong J., Guo Z., Liu J., Yang T.e., Qin C. (2019). Microstructure and properties of CVD coated Ti(C, N)-based cermets with varying WC additions. Int. J. Refract. Met. Hard Mater..

[41] Eray E., Boffa V., Jørgensen M.K., Magnacca G., Candelario V.M. (2020). Enhanced fabrication of silicon carbide membranes for wastewater treatment: From laboratory to industrial scale. J. Membr. Sci..

[42] Hashimoto R., Ito A., Goto T. (2015). Effect of deposition atmosphere on the phase composition and microstructure of silicon carbide films prepared by laser chemical vapour deposition. Ceram. Int..

[43] Chastain J., King R.C. (1992). Handbook of X-ray photoelectron spectroscopy. Perkin-Elmer Corp..

[44] You Q., Liu Y., Wan J., Shen Z., Li H., Yuan B., Cheng L., Wang G. (2017). Microstructure and properties of porous SiC ceramics by LPCVI technique regulation. Ceram. Int..

[45] Labropoulos A.I., Athanasekou C.P., Kakizis N.K., Sapalidis A.A., Pilatos G.I., Romanos G.E., Kanellopoulos N.K. (2014). Experimental investigation of the transport mechanism of several gases during the CVD post-treatment of nanoporous membranes. Chem. Eng. J..

[46] Wang C.-F., Tsai D.-S. (2000). Low pressure chemical vapor deposition of silicon carbide from dichlorosilane and acetylene. Mater. Chem. Phys..

[47] Alam J., Alhoshan M., Dass L.A., Shukla A.K., Muthumareeswaran M., Hussain M., Aldwayyan A.S. (2016). Atomic layer deposition of TiO_2_ film on a polyethersulfone membrane: Separation applications. J. Polym. Res..

[48] Sea B.-K., Ando K., Kusakabe K., Morooka S. (1998). Separation of hydrogen from steam using a SiC-based membrane formed by chemical vapor deposition of triisopropylsilane. J. Membr. Sci..

