# Supplementary material for: Effect of Long-Term Sodium Hypochlorite Cleaning on Silicon Carbide Ultrafiltration Membranes Prepared via Low-Pressure Chemical Vapor Deposition
Source: Membranes (Basel). 2024 Jan 15;14(1):22. doi: 10.3390/membranes14010022 (PMC10820315; doi:10.3390/membranes14010022)
Supplement: Supplementary file 1 [file membranes-14-00022-s001.zip › membranes-2795354-supplementary.pdf]

membranes

MDPI

Supplementary Materials

Effect of Long-Term Sodium Hypochlorite Cleaning on Silicon Carbide Ultrafiltration Membranes Prepared via Low-Pressure Chemical Vapor Deposition

Asif Jan [1,*], Mingliang Chen [1,2], Michiel Nijboer [2], Mieke Luiten-Olieman [2], Luuk Cornelis Rietveld [1] and Sebastiaan Gerard Jozef Heijman [1]

[1] Section of Sanitary Engineering, Department of Water Management, Faculty of Civil Engineering and Geosciences, Delft University of Technology, Stevinweg 1, 2628 CN Delft, The Netherlands; m.chen-1@tudelft.nl (M.C.); l.c.rietveld@tudelft.nl (L.C.R.); s.g.j.heijman@tudelft.nl (S.G.J.H.)

[2] Inorganic Membranes, MESA + Institute for Nanotechnology, University of Twente, 7500 AE Enschede, The Netherlands; m.p.nijboer@utwente.nl (M.N.); m.w.j.luiten@utwente.nl (M.L.-O.)

* Correspondence: a.jan@tudelft.nl

1. Thickness of SiC layer on silicon wafers at different deposition conditions measured by ellipsometry.

Table S1. SiC layer thickness as a function of different deposition conditions.

S.No.	Temperature (°C)	Pressure (mTorr)	Time (mins)	SiC Thickness (nm)
**1.**	750	600	60	16
**2.**	860	100	30	16

2. SiC-7-2A and SiC-8-2A membranes post-ageing.

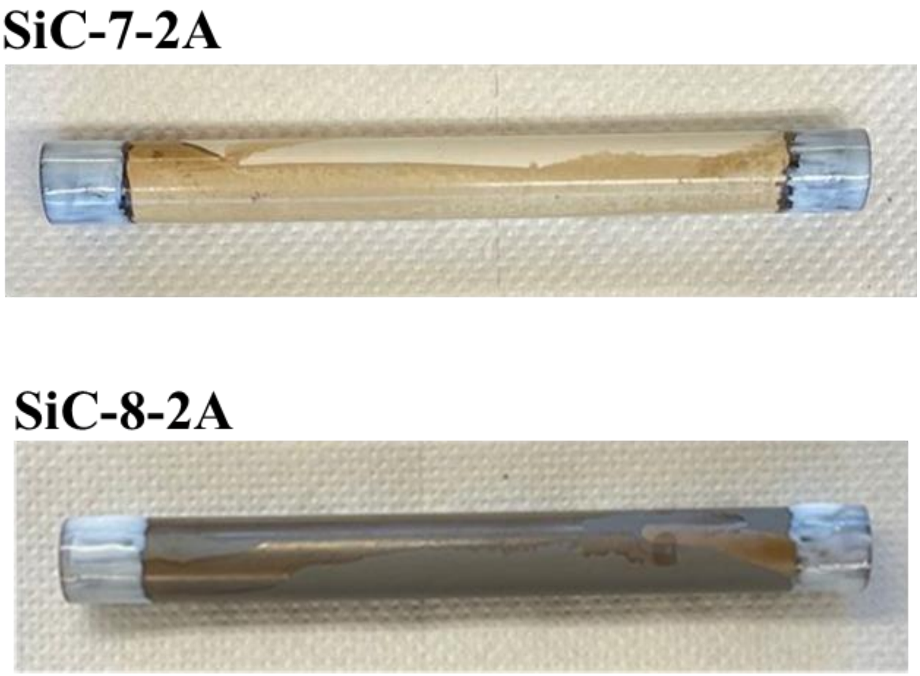

Figure S1. SiC membranes after ageing in NaClO for 200hrs.
